# Cellulose Nanofibers Prepared via Pretreatment Based on Oxone^®^ Oxidation

**DOI:** 10.3390/molecules22122177

**Published:** 2017-12-08

**Authors:** Chang-Qing Ruan, Simon Gustafsson, Maria Strømme, Albert Mihranyan, Jonas Lindh

**Affiliations:** Nanotechnology and Functional Materials, Department of Engineering Sciences, Uppsala University, Box 534, 75121 Uppsala, Sweden; changqing.ruan@angstrom.uu.se (C.-Q.R.); simon.gustafsson@angstrom.uu.se (S.G.); maria.stromme@angstrom.uu.se (M.S.); albert.mihranyan@angstrom.uu.se (A.M.)

**Keywords:** higher CNF production efficiency, Oxone^®^ oxidation, pretreatment, homogenization

## Abstract

Softwood sulfite bleached cellulose pulp was oxidized with Oxone^®^ and cellulose nanofibers (CNF) were produced after mechanical treatment with a high-shear homogenizer. UV-vis transmittance of dispersions of oxidized cellulose with different degrees of mechanical treatment was recorded. Scanning electron microscopy (SEM) micrographs and atomic force microscopy (AFM) images of samples prepared from the translucent dispersions showed individualized cellulose nanofibers with a width of about 10 nm and lengths of a few hundred nm. All results demonstrated that more translucent CNF dispersions could be obtained after the pretreatment of cellulose pulp by Oxone^®^ oxidation compared with the samples produced without pretreatment. The intrinsic viscosity of the cellulose decreased after oxidation and was further reduced after mechanical treatment. Almost translucent cellulose films were prepared from the dispersions of individualized cellulose nanofibers. The procedure described herein constitutes a green, novel, and efficient route to access CNF.

## 1. Introduction

Cellulose nanofibers (CNF) which possess properties such as high aspect ratio (L/d), large specific surface area, biodegradability, low coefficient of thermal expansion [1], great mechanical properties [2], and high gas barrier properties [3] are nano-sized fibers less than 100 nm wide produced from cellulose [4]. Due to their unique properties, CNF has been utilized in many areas such as tissue engineering [5], hydrogel contact lenses [6], filtration [7], electronic devices [8,9,10,11], carbon nanofiber production [12], drug delivery [13], wound healing [14], and translucent conducting films [15].

CNF are currently manufactured from several different sources of cellulose, where bleached sulfite softwood pulp is one of the most common sources [16]. A number of approaches, such as acid hydrolysis treatment, biosynthesis, and mechanical disintegration have been developed to produce CNF from cellulose, of which mechanical treatment is the main method of production [16]. However, the mechanical treatment methods e.g., grinding [17], electrospinning [18], and homogenizing are very energy consuming. To decrease the energy consumption of the cellulose fiber disintegration, pretreatment methods for cellulose are of vital importance where enzymatic and chemical pretreatment are the two main strategies employed.

In the case of chemical pretreatment, 2,2,6,6-tetramethylpiperidine-1-oxyl (TEMPO)-mediated oxidation and carboxymethylation have been frequently employed. Both of these pretreatment methods rely on introducing surface charges in the form of carboxyl groups on the cellulose fibers, which facilitates the fibrillation of the cellulose due to repulsive forces [3,19,20,21].

TEMPO-mediated oxidation and successive mild disintegration in water provides individual nanocellulose fibers. However useful the TEMPO-mediated oxidation is, providing low depolymerization and being mainly confined to the cellulose surface, some drawbacks are associated with the reaction; the TEMPO oxidation requires relatively strict control of the pH of the reaction solution and TEMPO often require stoichiometric amounts of halogen-containing co-oxidants (e.g., TEMPO/NaBr/NaClO) to mediate the oxidation reaction [22], which should be avoided in industrial processes [23]. Further, an additional possible drawback of the TEMPO-mediated process is the risk of having trace amounts of reactive radicals in the product, which might limit the usefulness of the material in e.g., bio-applications [24]. Based on the mentioned drawbacks of the TEMPO-mediated oxidation, there is room for alternative oxidation methods to complement TEMPO-mediated oxidation. Oxone^®^ oxidation is a promising alternative, which might serve as a complement to TEMPO-mediated oxidation.

Oxone^®^ is the trade name of the stable triple salt with the chemical formula 2KHSO_5_·KHSO_4_·K_2_SO_4_, containing potassium peroxymonosulfate (KHSO_5_) as the active component, which is a salt of Caro’s acid (H_2_SO_5_). Recently, we reported that the C6 hydroxyl groups of cellulose could be oxidized to carboxylic groups by Oxone^®^ in water [25] to provide anionic groups on the surface of cellulose nanofibers. The charges on the oxidized cellulose nanofibers enhance the swelling of the cellulose by electrostatic repulsion to facilitate the fibrillation into nanofibers [26]. Although lower carboxylic acid content has so far been achieved via Oxone^®^ oxidation in comparison to TEMPO-mediated oxidation, significant amounts of carboxylic acid groups can be obtained, which should facilitate the fibrillation of pulp to nanofibers.

In this work, for the first time, cellulose nanofibers from softwood sulfite bleached pulp were produced by pretreatment with Oxone^®^ oxidation followed by high-shear homogenization. The data collected from UV-vis spectroscopy, in addition to images from SEM and atomic force microscopy (AFM), demonstrated that with the same homogenizing treatment, cellulose pulp oxidized by Oxone^®^ and with higher carboxylic acid content was more easily fibrillated than untreated pulp with a lower carboxylic acid content.

## 2. Results and Discussion

The Oxone^®^ oxidation of pulp was conducted according to a recently developed method [25]. In this work, 4% (*w*/*v*) concentration of cellulose pulp was employed, which improved the oxidation efficiency. In fact, the carboxylic acid content for both pulp oxidized by Oxone^®^ alone (pulp-O) and by Oxone^®^ in combination with NaBr (pulp-O-Br) (see Table 1) increased, compared with the carboxylic acid content in previously published work [25]. In order to investigate the effect of NaBr on the oxidation, NaBr was employed with Oxone^®^ oxidation at room temperature. Pristine softwood sulfite bleached cellulose pulp (pulp) was used as a control. As can be seen in Table 1, the oxidation performed at 80 °C (pulp-O) provided the highest carboxylic acid content.

Pulp-O was prepared at 80 °C under stirring, while pulp-O-Br was produced at room temperature with the addition of NaBr under stirring. At the beginning of the oxidation reaction, the magnetic stirrer rotated slowly due to the high viscosity of the slurry. About three hours later, the stirrer could rotate fluently both in the reaction mixtures with and without NaBr indicating that the intrinsic viscosity of the dispersion had decreased significantly, probably due to degradation of the cellulose pulp.

**Figure 1:**
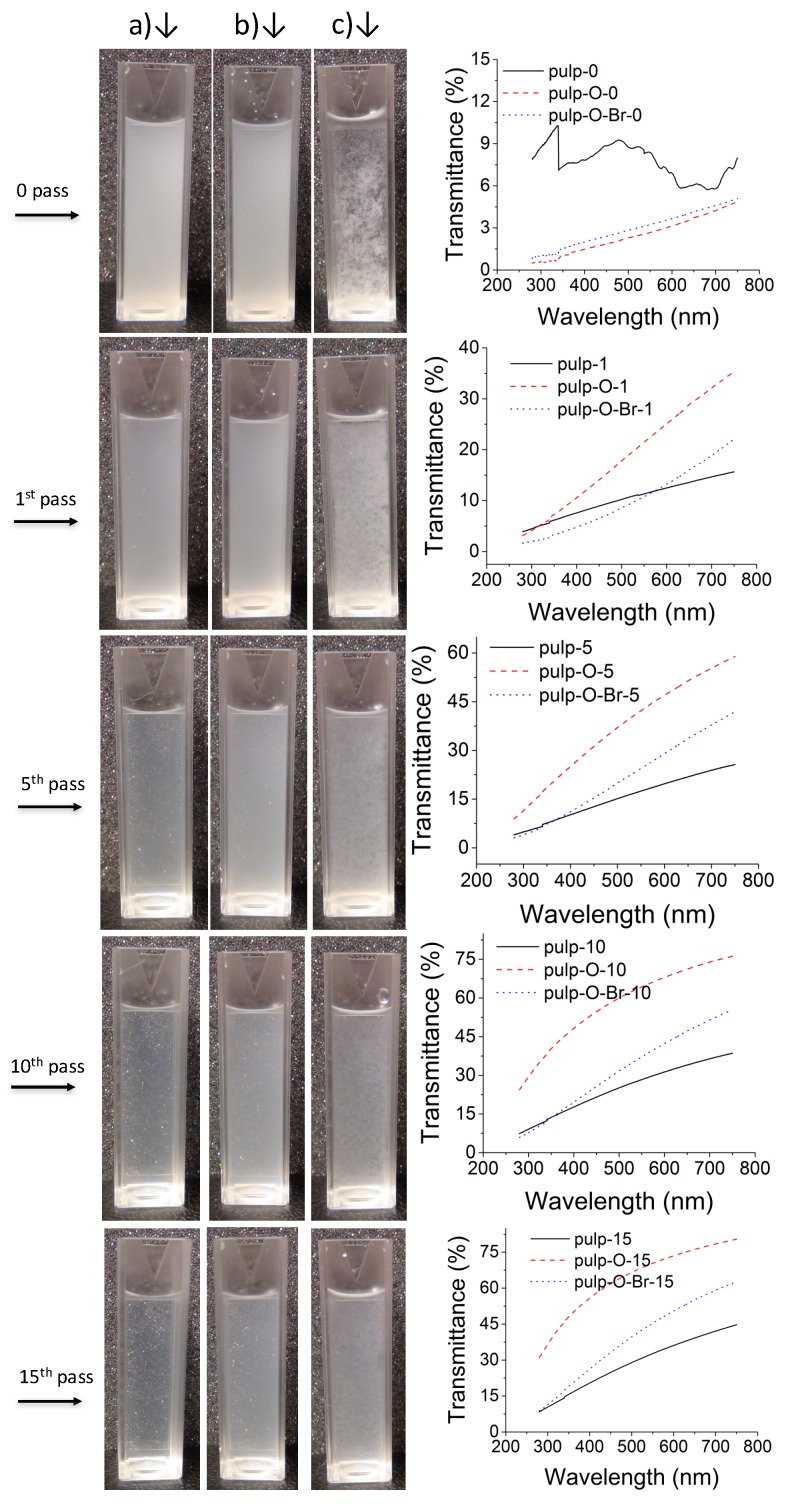
Photographs of dispersions (0.1%, *w*/*v*) of (**a**) pulp-O; (**b**) pulp-O-Br; and (**c**) pulp after different numbers of passes through a homogenizer and the corresponding UV-vis transmittance spectra at a wavelength range from 285 to 750 nm.

The purified and never-dried pulp-O, pulp-O-Br, and pulp were dispersed in deionized water to obtain diluted dispersions at a concentration of 0.1% (*w*/*v*), which were then disintegrated with a high-shear homogenizer. Each dispersion was passed through the homogenizer 15 times. At the 1st, 5th, 10th and 15th pass, 3 mL aliquots were withdrawn and transferred into a disposable cuvette (Figure 1). The dispersions of pulp-O, pulp-O-Br, and pulp without mechanical treatment were used as a control. Figure 1 shows that all three dispersions of pulp-O, pulp-O-Br, and pulp became more dispersed and translucent as the number of passes through the homogenizer increased. The dispersion of pulp-O was observed to be the most translucent after 15 passes (Figure 1a). These results indicate that the oxidized cellulose pulp (both pulp-O and pulp-O-Br) with its repulsive charges is more easily fibrillated and requires less mechanical treatment to form translucent dispersions.

The transmittance of all the dispersions was measured from 285 to 750 nm in a quartz cuvette by a UV-vis spectrometer. The UV-vis spectra in Figure 1 show the comparison of the transmittance between different dispersions after a certain number of passes. It is obvious that in most cases, the dispersion of pulp-O had the highest transmittance, which also correlates to the sample having the highest content of carboxylic groups and hence being the most facile to fibrillate. As the dispersion of pulp was prone to precipitate quickly, the UV-vis spectrum of pulp fluctuates and the transmittance was overestimated at some wavelengths. The transmittance values at 550 nm in Table 1 shows that the disintegration of pulp-O was the most efficient and pulp-O also had the highest carboxylic acid content, 1.073 mmol/g. The UV-vis data confirm the results shown in the images in Figure 1, that the dispersion of pulp-O after 15 passes was the most translucent and the easiest to disintegrate.

The intrinsic viscosities of pulp and oxidized pulps with and without mechanical treatment (15 passes) were measured with a viscometer. The intrinsic viscosity values of the samples are shown in Table 1. The intrinsic viscosity of oxidized cellulose decreased compared to unoxidized cellulose pulp, which indicates that the cellulose pulp degraded during the oxidation process. The acidic reaction solution (pH at about 2) and 80 °C reaction temperature might induce hydrolysis of the cellulose pulp during the oxidation. Mechanical treatment with a homogenizer further reduced the intrinsic viscosity of all samples.

**Figure 2:**
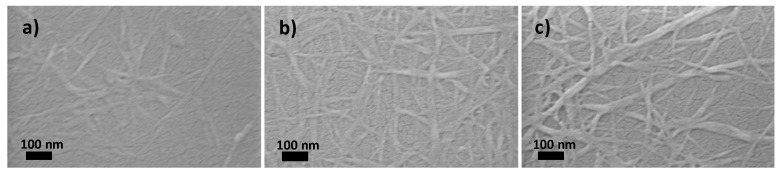
SEM images of dispersions of (**a**) pulp-O; (**b**) pulp-O-Br; and (**c**) pulp after 15 passes through a homogenizer.

**Figure 3:**
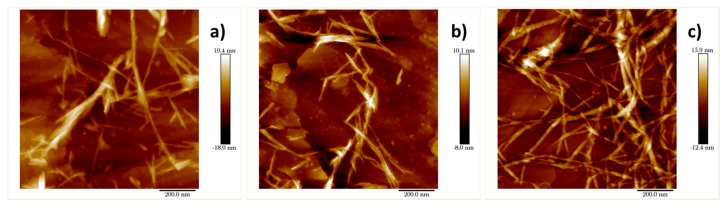
Atomic force microscopy (AFM) images of dispersions of (**a**) pulp-O; (**b**) pulp-O-Br; and (**c**) pulp after 15 passes through a homogenizer.

The SEM and AFM images of dispersions of pulp-O, pulp-O-Br, and pulp after 15 passes are shown in Figure 2 and Figure 3. CNF could be observed in all these dispersions, which indicates that cellulose nanofibers were produced after a certain amount of mechanical treatment with a homogenizer. In Figure 2a,b and Figure 3a,b, almost all of the cellulose fibers had disintegrated into nanofibers with a diameter of about 10 nm, while cellulose fibers with a diameter of about 50 nm were still present in Figure 2c and Figure 3c. Some bundles of CNF could be observed in Figure 3a,b, which might be due to aggregation during the drying of dispersions on the mica wafers. When comparing Figure 2a,b and Figure 3a,b, more aggregated nanofibers could be seen in Figure 2b and Figure 3b, which might be due to the fact that pulp-O-Br had less surface charge. The results from both SEM and AFM indicate that after the same treatment with the homogenizer, the pulp-O with the highest carboxylic acid content could most easily be disintegrated, which means pulp-O requires less mechanical treatment to obtain a certain degree of dispersion compared with the pulp-O-Br and pulp. Pretreatment with Oxone^®^ oxidation makes the fibrillation process more efficient and less energy demanding, although it decreases the aspect ratio of the nanofibers.

**Figure 4:**
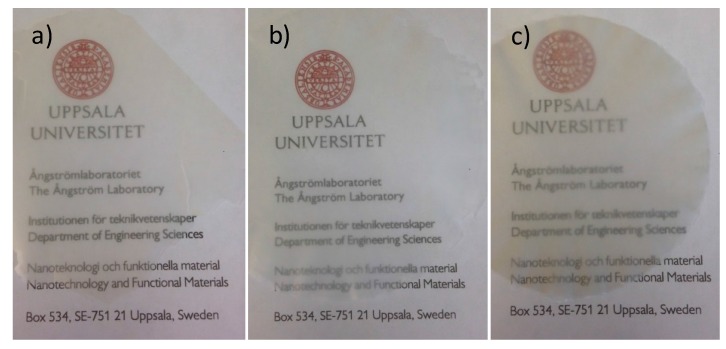
Photographs of translucent films of (**a**) pulp-O; (**b**) pulp-O-Br; and (**c**) pulp after 15 passes through the homogenizer. The films were photographed on top of a printed page to demonstrate their transparency.

The photographs of prepared CNF films in Figure 4 show that the three films are translucent to different degrees, which indicate that the homogenized Oxone^®^-oxidized and unoxidized cellulose pulp have different potential for forming translucent films. The three films had different thicknesses, (film of pulp-O 9 µm, pulp-O-Br 28 µm; and pulp 37 µm) and the thickness was hard to control (as shorter and thinner pulp-O nanofibers are more prone to penetrate the filter paper of 0.65 µm pore size used, less pulp-O nanofibers were left to form the film). The absorbance of the films was measured at 550 nm and correlated to the thickness of the films by the ratio of the percent light absorbed and the thickness of the films in µm to give the following values: pulp-O 1.33 abs%/µm, pulp-O-Br 1.46 abs%/µm, and pulp 2.43 abs%/µm. Hence, the absorbance values for the films were in agreement with the data from the transmittance measurements of the dispersions.

## 3. Materials and Methods

### 3.1. Materials

Never-dried cellulose bleached sulfite softwood dissolving pulp was supplied by Domsjö Fabrikerna AB, Örnsköldsvik, Sweden. Oxone^®^ (monopersulfate compound, 2KHSO_5_·KHSO_4_·K_2_SO_4_); sodium bromide (NaBr) and other chemicals were of reagent or analytical grade and were used as received from commercial suppliers. Deionized water was used throughout all experiments.

### 3.2. Oxone*^®^* Oxidation

Oxone^®^ oxidation of cellulose pulp was conducted as described in a previously published report [25] with minor modifications. Briefly, never-dried cellulose pulp (corresponding to a dry weight of 2 g) was dispersed in 40 mL deionized water by thorough high-energy ultrasonication (VibraCell 750 W, Sonics, Newtown, CT, USA). The pulp dispersion was added into a round-bottom flask containing 9.1 g Oxone^®^ (2.4 equivalents per anhydroglucose unit) pre-dissolved in 10 mL deionized water. The Oxone^®^ oxidation was performed at 80 °C under stirring with the flask sealed by a stopper. After 24 h, the Oxone^®^-oxidized pulp (pulp-O) was thoroughly washed with deionized water by filtration and stored in a wet state at 4 °C for further treatment and analysis.

Oxone^®^ oxidation with NaBr of cellulose pulp was carried out at room temperature. The procedure was similar to the procedure above, but with the addition of NaBr (0.636 g, 0.5 equivalents per anhydroglucose unit) to the dispersion of pulp and Oxone^®^. The purified sample was named pulp-O-Br.

The carboxylate content of pulp-O and pulp-O-Br were determined by an electric conductivity titration method [27]. The recovery yields of pulp-O and pulp-O-Br were over 80% and 85%, respectively.

### 3.3. Mechanical Disintegration

The dispersions of cellulose pulp, pulp-O, and pulp-O-Br in water (150 mL) at 0.1% (*w*/*v*) were homogenized with a Microfluidizer LM20 (Microfluidics, Westwood, MA, USA) at 1000 bar with 15 passes. Photographs of the slurries of cellulose pulp, pulp-O, and pulp-O-Br, and their treated samples after 1, 5, 10, and 15 passes were taken during the fluidizing process. 

### 3.4. Optical Transmittance

The slurries of cellulose pulp, pulp-O, and pulp-O-Br, and their treated samples after 1, 5, 10, and 15 passes were introduced into a quartz cuvette (SUPRASIL^®^300), and the transmittances were measured in a wavelength range of 750–285 nm with a Shimadzu UV-vis spectrometer (UV-1800). The spectrum of a cuvette filled with deionized water was used as a reference and to calibrate the transmittance of the samples.

### 3.5. Preparation of CNF Films

The suspensions of cellulose pulp, pulp-O, and pulp-O-Br obtained after 15 passes at 1000 bar in the homogenizer were drained over a nylon filter (Durapore; 0.65 μm DVPP; Merck Millipore, Burlington, MA, USA) under suction. The wet cellulose fibers on the filter paper were then transferred to dry in a hot-press (80 °C) (Rheinstern, Germany) to obtain a CNF film.

### 3.6. Scanning Electron Microscopy (SEM)

Scanning electron micrographs were recorded with a LEO1550 field-emission SEM instrument (Zeiss, Oberkochen, Germany) operated at an accelerating voltage of 500 V with an in-lens secondary electron detector. The suspensions of cellulose pulp, pulp-O, and pulp-O-Br obtained after 15 passes at 1000 bar through the homogenizer were diluted and dropped onto silicon wafers. The silicon wafers were dried at ambient temperature and atmosphere overnight. Samples were sputtered with gold/palladium for 40 s to avoid charging effects during SEM detection.

### 3.7. Atomic Force Microscopy (AFM)

A Bruker Dimension Icon AFM system (Billerica, MA, USA) with a Bruker silicon nitride SCANASYST-AIR probe, which had symmetric pyramid geometry with a nominal tip radius of 2 nm, was used to obtain the images. The suspensions of cellulose pulp, pulp-O, and pulp-O-Br were obtained after homogenization using 15 passes at 1000 bar. The solutions were diluted and dropped onto mica wafers, which were dried at ambient temperature and atmosphere and then mounted on magnetic holders with a double-sided adhesive tape. The images were acquired under the peak-force tapping mode, using the manufacturer’s ScanAsyst automatic optimization algorithm. Post-processing of the pictures was performed using Bruker NanoScope Analysis v. 1.6 software (Billerica, MA, USA).

### 3.8. Intrinsic Viscosity

The intrinsic viscosity was determined for the samples according to the literature with minor modifications [28]. Briefly, cellulose samples were dispersed thoroughly in 7.5 mL deionized water in a 15-mL centrifugation tube, and the dispersions were then purged with nitrogen. Bis(ethylenediamine)copper(II) hydroxide solution (7.5 mL, 1.0 M in H_2_O) was mixed without stopping the continuous nitrogen purging. The tube was capped tightly and shaken vigorously with a mechanical shaker to make the cellulose samples dissolve thoroughly. The solution was added to the viscometer (Ubbelohde viscometer, SI Analytics GmbH, Weilheim, Germany) and was allowed to equilibrate for >5 min at 25.0 °C in a water bath. Measurements were repeated five times for each sample and all measurements were performed at 25.0 °C. The experiments were recorded by a video camera and a digital timer for accurate timing. Intrinsic viscosities of the samples were calculated according to the ASTM standard method D1795-96.

## 4. Conclusions

In this study, cellulose nanofibers were prepared by pretreatment with Oxone^®^ oxidation followed by mechanical treatment using homogenization. Pretreatment with Oxone^®^ oxidation of cellulose pulp could make the subsequent mechanical treatment more efficient and lower the energy consumption, which was demonstrated by the translucent films produced, the UV-vis data, SEM and AFM images. The intrinsic viscosity values of cellulose decreased due to degradation accompanying the oxidation process. Translucent cellulose films were prepared from CNF dispersions, which are of potential interest in a number of applications such as packaging applications, printed electronics, and diagnostics.

## Figures and Tables

**Table 1:** Physicochemical properties of samples.

Sample	pulp	pulp-15	pulp-O	pulp-O-15	pulp-O-Br	pulp-O-Br-15
Intrinsic viscosity (η) (dL/g)	2.01	1.149	0.375	0.294	0.510	0.456
Transmittance at 550 nm (%)	8.37	32.69	2.67	70.34	3.23	45.31
Carboxylic acid content (mmol/g)	0.077	/	1.073	/	0.585	/

Samples passed through the homogenizer 15 times are denoted with the suffix −15.
